# Supplementary figures and images for: OrganoidTracker: Efficient cell tracking using machine learning and manual error correction
Source: PLoS One. 2020 Oct 22;15(10):e0240802. doi: 10.1371/journal.pone.0240802 (PMC7580893; doi:10.1371/journal.pone.0240802)

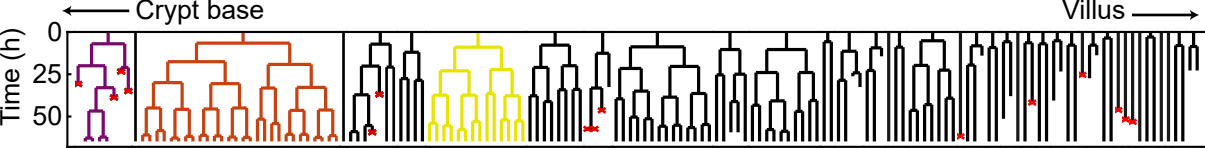

Supplement: S1 Fig — Red crosses denote the positions where a cell death was observed in the microscopy images. This figure will also become part of an upcoming publication. (PDF) [file pone.0240802.s001.pdf]
