# Supplementary material for: OrganoidTracker: Efficient cell tracking using machine learning and manual error correction
Source: PLoS One. 2020 Oct 22;15(10):e0240802. doi: 10.1371/journal.pone.0240802 (PMC7580893; doi:10.1371/journal.pone.0240802)
Supplement: S1 Appendix — Explanation and equations of the scoring system used to determine whether a given nucleus is a mother cell. (PDF) [file pone.0240802.s005.pdf]

Cell division scoring system

The fluorescent H2B-mCherry marker labels the histones, which normally results in the entire nucleus being observed. However, during a cell division the nuclear envelope breaks down and instead we observe the compacted chromosomes. It would therefore not be correct to say that we observe the nucleus during a cell division, as there is no nucleus during certain parts of the division. However, to make the text easier to read we will still call these compacted chromosome structures "nuclei" in the following text, instead of constantly writing "nuclei or compacted chromosome structures".

The scoring system takes into account both the mother cell and two daughter cells. The scoring system is designed such that lower values are more optimal; the score can therefore be considered as a penalty score or an energy. If the score is above -1, the possibility of a cell division is not even considered by the min-cost flow solver.

As stated in the main text, the linking algorithm starts by defining all possible links, which are the nearest-neighbor links along with links that span a distance at most twice as long as the distance to the nearest neighbor. Division scores are calculated for every case where a nucleus at one time point has at least two possible links to the next time point. If there are three or more possible links, then there are also three or more possible daughters. As biologically only two daughter cells are possible, scores are calculated for all possible combinations of two daughter cells and the lowest score is chosen.

Every potential mother cell gets a score using the following equation:

$$\begin{aligned}\texttt{score} = & \texttt{mother\_intensity\_delta} + \texttt{daughter\_intensity\_difference} \\ & + \texttt{daughter\_intensity\_delta} + \texttt{mother\_volume} \end{aligned} \tag{1}$$

Of these scores, `mother_volume` carries the heaviest weight. By default, it is 10 which is so high that it makes it impossible for the min-cost flow solver to create a division here. Only if the volume of the mother nucleus is almost as large as the volume of the new daughter cells combined, the score becomes 0 which allows for a division.

$$\texttt{mother\_volume} = \begin{cases} 0 & \text{if } V_{mother}/(V_{daughter1} + V_{daughter2}) > 0.95 \\ 10 & \text{otherwise} \end{cases}$$

The other rules are then used to pick the most likely division candidate, if multiple possibilities of nuclei show such a volume change. The next rule makes use of the observation that the daughter nuclei are located at a different position than the mother nucleus was. Therefore, the fluorescence intensity at the location of the mother cell before the division should have decreased after the division. For non-dividing nucleus, it is rare that nuclei move this rapidly, so non-dividing nuclei will get a lower score.

$$\texttt{mother\_intensity\_delta} = \begin{cases} -1 & \text{if } I_{current}/(I_{next} + 0.0001) > 2 \\ 0 & \text{if } 2 \geq I_{current}/(I_{next} + 0.0001) > 1.4 \\ 1 & \text{otherwise} \end{cases}$$

Here, $I_{current}$ is the average pixel intensity at the location of the mother nucleus at the last time point where a single nucleus was visible, with the intensities of the image normalized from 0 to 1. The location of the mother nucleus is derived from the Gaussian fit: we look at all pixels at the XY plane of the nucleus center where the Gaussian fit still has 20% of its maximum intensity. I_{next} is the intensity at exactly the same pixel locations in the next time point. The $+0.0001$ is used to prevent a division by zero for the rare cases where the image becomes completely black at the location where the mother location was.

For the daughter nuclei the opposite should happen: where there was no nucleus before, there now is a nucleus. The intensity should therefore go up. For a single daughter, the score is calculated as follows:

$$\texttt{daughter1\_intensity\_delta} = \begin{cases} -1 & \text{if } I_{current}/(I_{prev} + 0.0001) > 2 \\ 0 & \text{if } 2 \geq I_{current}/(I_{prev} + 0.0001) > 1 \\ 1 & \text{otherwise} \end{cases}$$

Here, $I_{current}$ is the intensity of the daughter cell nucleus, in the first time point where the two daughter nuclei were visible. Again, the Gaussian fit is used to find out which pixels are inside the daughter cell nucleus, in exactly the same way as this was done for the mother nucleus. I_{prev} is the intensity at the same pixel locations, but for the previous time point.

For daughter nucleus 2 the same equation is used. Both values are then summed:

$$\texttt{daughter\_intensity\_delta} = -1 + \texttt{daughter1\_intensity\_delta} + \texttt{daughter2\_intensity\_delta}$$

1 is subtracted here to relax the scoring system and make divisions more likely.

Finally, we expect both daughter nuclei to have the same intensity, as they were created out of a single nucleus minutes ago. The only deviations of intensity between the two nuclei should occur if one daughter nucleus ends up further away from the microscope objective than the other. Therefore, we can lower the score for any difference in the average intensity of the two:

$$\texttt{daughters\_intensity\_difference} = \frac{|I_{daughter1} - I_{daughter2}|}{2}$$

Again, the intensity is the average intensity of the pixels within the nucleus, as defined by the Gaussian fit.
