# Supplementary material for: OrganoidTracker: Efficient cell tracking using machine learning and manual error correction
Source: PLoS One. 2020 Oct 22;15(10):e0240802. doi: 10.1371/journal.pone.0240802 (PMC7580893; doi:10.1371/journal.pone.0240802)
Supplement: Attachment — Submitted filename: Response to Reviewers.docx [file pone.0240802.s007.docx]

Response to Reviewers

## Reviewer #1:

The authors have done a good job of clarifying the relationship between this work and past work, as well as describing the particular challenges of organoid images. I wish the discussion in the text had the level of detail of the "response to reviewers," though I realize that this would perhaps have been distracting. I support the publication of the paper.

We thank the reviewer again for taking the time to review our paper, and we are happy that the reviewer supports the publication of the manuscript.

## Reviewer #2:

The authors have addressed all questions, issues and suggestions of this
reviewer in their revised manuscript (and I thank you for that). The
manuscript is now even more clear. Well done. The changed parts of the
manuscript align well in the manuscript.


If I were to suggest final set of changes, I would ask for the following minor
improvements or clarifications:

Regarding the requested comparison to the TrackMate and Mamut, I thank the
authors for doing this extra work and for sharing with me the new findings.
I would like to encourage them to actually include this piece of
information into the manuscript. Not only it shows another "plus points"
for the SW of the authors, I think it is indeed a fair (despite "only"
quantitative) comparison of SW that include the same stages (detection,
linking _and_ curation) -- and that's useful piece of information also for
the community.

We have followed the suggestions of the reviewer and added a section ‘Comparison with other methods’ to the manuscript (lines **421-452** in the file with tracked changes**)**, describing our cell detection and linking results for TrackMate. Briefly, for cell detection TrackMate displayed a low recall for our test data set of 8 organoids – on average, one third of all cells in an image are not detected. This relatively poor result makes linking challenging, which we confirmed when we attempted linking using the LAP tracker of TrackMate with default settings.

Regarding the conversion of nuclei detection (blobs) into
pseudo-segmentation masks (by placing spheres of 5 um radius), why were
some detections (and seg. masks) removed? It biases the measurement, no?
Please clarify in the manuscript if the filtering was due to the fact the only six
time points are fully annotated (in which case the filtering makes sense).
What is the average diameter of nuclei, is it less than 10 um? Otherwise,
the spheres are over-segmenting and bias the DET score.

We indeed filtered only because six time points are fully annotated. The text has been edited to clarify this, see lines **223-235** in the manuscript with tracked changes.

In less crowded areas we think a sphere of 10 µm is, at least on average, an accurate representation of a nucleus. However, in crowded regions the nuclei are often ellipsoidal. In those regions, the nuclei are arranged in a closely-packed epithelium, with the long dimension larger than 10 μm and the short dimension smaller. This results in a risk of oversegmentation, resulting from overlapping spheres. We prevented oversegmentation by assigning each pixel in the overlapping region of two spheres to the nearest nucleus center. As a result, the masks no longer end up as spherical, but actually start to look like the actual mask of the nucleus.

In Fig 2, perhaps the first parentheses on line 2 could be "(obtained from
manual detection)"... detection is less difficult activity than tracking,
while it is enough for creating training data for the network... just an idea.

We thank the reviewer for this suggestion, and have implemented it.

Line 8 (of the document with marked changes): "the the historical"
Line 48: "in in time-lapse movies."

We thank the reviewer for spotting these errors, and have fixed them.

PS: I apologize to the first author for mixing up his first name and surname
(in my first review I wrongly wrote "Rutger et al.").
